# Male involvement in birth preparedness and complication readiness for emergency obstetric referrals in rural Uganda

**DOI:** 10.1186/1742-4755-8-12

**Published:** 2011-05-07

**Authors:** Othman Kakaire, Dan K Kaye, Michael O Osinde

**Affiliations:** 1 Department of Obstetrics and Gynecology, School of Medicine, Makerere University College of Health Sciences, P.O. Box 7072, Kampala, Uganda; 2 Department of Obstetrics and Gynecology, Kabale Regional Hospital, P.O.B ox 7, Kabale, Uganda

## Abstract

**Background:**

Every pregnant woman faces risk of life-threatening obstetric complications. A birth-preparedness package promotes active preparation and assists in decision-making for healthcare seeking in case of such complications. The aim was to assess factors associated with birth preparedness and complication-readiness as well as the level of male participation in the birth plan among emergency obstetric referrals in rural Uganda.

**Methods:**

This was a cross-sectional study conducted at Kabale regional hospital maternity ward among 140 women admitted as emergency obstetric referrals in antenatal, labor or the postpartum period. Data was collected on socio-demographics and birth preparedness and what roles spouses were involved in during developing the birth plan. Any woman who attended antenatal care at least 4 times, received health education on pregnancy and childbirth danger signs, saved money for emergencies, made a plan of where to deliver from and made preparations for a birth companion, was deemed as having made a birth plan. Multivariate logistic regression analysis was conducted to analyze factors that were independently associated with having a birth plan.

**Results:**

The mean age was 26.8 ± 6.6 years, while mean age of the spouse was 32.8 ± 8.3 years. Over 100 (73.8%) women and 75 (55.2%) of their spouses had no formal education or only primary level of education respectively. On multivariable analysis, Primigravidae compared to multigravidae, OR 1.8 95%CI (1.0-3.0), education level of spouse of secondary or higher versus primary level or none, OR 3.8 95%CI (1.2-11.0), formal occupation versus informal occupation of spouse, OR 1.6 95%CI (1.1-2.5), presence of pregnancy complications OR 1.4 95%CI (1.1-2.0) and the anticipated mode of delivery of caesarean section versus vaginal delivery, OR 1.6 95%CI (1.0-2.4) were associated with having a birth plan.

**Conclusion:**

Individual women, families and communities need to be empowered to contribute positively to making pregnancy safer by making a birth plan.

## Introduction

Maternal mortality remains a public health challenge worldwide, and the global maternal mortality ratio of 342, 900/100,000 live births annually is still unacceptably high [1]. A disproportionately high burden of these maternal deaths is borne by developing countries such as Uganda, with a maternal mortality ratio of 432 per 100,000 live births [2]. These deaths arise from pregnancy, childbirth or postpartum complications. A key strategy that can reduce the number of women dying from such complications is making a birth plan that constitutes birth-preparedness and complication-readiness measures for pregnant women, their spouses and their families [3]. Birth-preparedness and complication-readiness is a comprehensive package aimed at promoting timely access to skilled maternal and neonatal services. The birth-preparedness package promotes active preparation and decision-making for delivery by pregnant women and their families [3]. This stems from the fact that every pregnant woman faces risk of sudden and unpredictable life threatening complications that could end in death or injury to herself or to her infant [4].

The key elements of the birth plan package include recognition of danger signs, a plan for a birth attendant, a plan for the place of delivery, and saving money for transport or other costs in case the need arises [4]. In addition, for birth preparedness, a potential blood donor and a decision-maker (in case of emergencies) need to be identified [5]. This is because complications such as hemorrhage are unpredictable and highly fatal if timely treatment is not obtained. This makes this package a very important strategy in developing countries, where obstetric services are poor. The obstetric referrals are women of low status and contribute significantly to maternal and neonatal morbidity and mortality [5].

In sub-Saharan Africa, pregnancy and childbirth continue to be viewed as solely a woman's issues [4]. A male companion at antenatal care is rare and in many communities, it is unthinkable to find male companions accompanying a woman to the labour room during delivery [6] However, men have social and economic power, especially in Africa, and have tremendous control over their partners. They decide the timing and conditions of sexual relations, family size, and whether their spouse will utilize available health care services [7]. Hence this situation makes male partner involvement critical if improvement in maternal health and reduction of maternal morbidity and mortality is to be realized. Strategies for involving men in maternal health services should aim at raising their awareness about emergency obstetric conditions, and engaging them in birth preparedness and complication readiness [4]. Male involvement will enable men to support their spouses to utilize emergency obstetric services early and the couple would adequately prepare for birth and ready themselves for complications. This would lead to a reduction in all three phases of delay [8] and thereby positively impact birth outcomes.

Kabale District is a rural highland district in southwestern Uganda, about 560 km from the capital city, Kampala. The topography is mainly interlocking and heavily cultivated hills with deep valleys. The three counties of Rubanda, Rukiga and Ndorwa, together with Kabale Municipality form Kabale District for which the 2002 national census estimated a population of 471,800, and an annual population growth rate of 3%. Kabale Regional Hospital is a public hospital, funded by the Uganda Ministry of Health, with a bed capacity of 200. The study was conducted at the obstetric unit of Kabale hospital, which serves as the major referral centre for other public and private hospitals within Kabale district, southwestern Uganda. In addition to providing emergency obstetric services to women referred from other centres, the hospital also provides antenatal care and delivery services for both low and high-risk pregnant women. Although patients are not expected to pay for their services, in emergency situations, they are managed within the means of existing resources, and often have to purchase sundries and their own medication. One consultant obstetricians, 2 registrars and 12 midwives ran the obstetric units of the hospital during the reviewed period. This study aim was to assess factors associated with birth preparedness and complication readiness as well as the level of male participation in the birth plan and healthcare seeking for emergency obstetric referrals in rural Uganda.

## Methods

### The study design

This was a cross-sectional study conducted at Kabale regional hospital maternity ward from July to October 2010. The study inclusion criteria were having been admitted to the hospital as an emergency referral in antenatal, labor or the postpartum period and willingness to consent for participation in the study. Participants were followed up to their discharge from hospital or death.

Research assistants who were health workers attached to the maternity ward recruited the participants. Using an interviewer-administered questionnaire, data was collected from 140 women on i) Socio-demographic variables such as age, education level, marital status (single versus married) and employment status ii) Medical history such as antenatal care attendance, obstetric history, reasons for referral, obstetric complications and care received and mode of delivery were recorded; iii) Birth preparedness, based on the number of arrangements a woman had made, including purchasing a safe delivery kit, saving money for delivery, arranging for transportation to delivery and making an emergency plan or looking for someone to take care of the household during hospitalization. (Any woman who attended antenatal care at least 4 times, received health education on pregnancy and childbirth danger signs, saved money for emergencies, made a plan of where to deliver from and made preparations for a birth companion, was deemed as having made a birth plan); iv) Data was also collected on what extent and in what roles spouses were involved in the birth plan. From review of the medical records, data was collected on the obstetric complications, reasons for referral, obstetric care obtained at the referral and referring sites and availability of a birth plan.

Data was analyzed using the STATA software (Release 9) to provide frequencies and percentages for categorical variables and means and standard deviations for numerical variables. At bivariate analysis, characteristics of the participants who had a birth plan were compared with those who did not have a birth plan, using Pearson's chi-square test for categorical data and Student t-test for numerical data. Multivariate logistic-regression analysis was conducted to analyze factors that were independently associated with having a birth plan, while assessing for interaction and collinearity. During the stepwise modeling for regression analyses, all variables of clinical importance or with p-value 0.2 and less on bivariate analysis were considered for inclusion. Ethical clearance was obtained from Kabale Regional Hospital. Participants were recruited after getting informed consent, at a time when they had recovered from the acute obstetric complications that necessitated their admission.

## Results

During the study period, 140 women were recruited from 158 eligible participants; 12 were too unwell to consent and 6 declined invitation to participate. Table 1 shows the socio-demographic characteristics and indications for referral for the 140 women. The mean age was 26.8 ± 6.6 years, while mean age of the spouse was 32.8 ± 8.3 years. Over 100 (73.8%) women and 75 (55.2%) of their spouses had no formal education or only primary level of education respectively; and 114 (81.8%) were in marital relationships. Table 2 shows the indication for referral as an obstetric emergency. Of the 140 women admitted as obstetric emergencies, the indication for admission was obstructed labor for 63 (45.0%) and fetal distress for 15 women (10.5%) and 7 (5.0%) with ruptured uterus.

**Table 1:** Table of socio-demographic characteristics (N = 140)

Characteristic	Mean ± standard deviation
*Age	26.8 ± 6.6

*Parity	3.3 ± 2.3

*Gestation age	38.1 ± 3.4

*Age of spouse	32.8 ± 8.3

*Education level*	
None	24 (17.7)
Primary	78 (56.1)
Secondary	30 (21.6)
Post-secondary	7 (5.0)

*Marital status*	
Single	25 (18.2)
Married	114 (81.8)

*Education level of spouse*	
None	8 (5.9)
Primary	67 (49.3)
Secondary	45 (33.1)
Post-secondary	20 (12.0)

*Occupation*	
Formal employment	20 (14.1)
Informal or no employment	121 (85.9)

Key: * Mean ± Standard Deviation

**Table 2:** Reason for referral (N = 140)

Characteristic	Number (Percentage)
*†Reason for referral*	
Anemia	2 (1.5)
Eclampsia	3 (2.1)
Fetal distress (including 5 with cord prolapse)	15 (10.5)
Hemorrhage	16 (18.4)
Obstructed or prolonged labor (including impending uterine rupture with IUFD)	63 (45.0)
Poor obstetric history	4 (2.9)
Malpresentation (including arm prolapse)	5 (3.6)
Multiple pregnancy (3 with retained twin)	6 (4.2)
Previous caesarean section	15 (10.5)
Ruptured uterus	4 (2.9)
Preterm labour	7 (5.0.)
Postpartum hemorrhage	4 (2.9)

*Referring Unit*	
Hospital	92 (67.3)
Health centre	13 (9.0)
Private clinic	34 (22.7)

Key: † Reason for referral identified at admission

Table 3 shows the birth preparedness for emergency complications and the role played by the husband. Sixty two women (44.3%) made savings for any eventuality such as pregnancy complications, 60 (42.9%) were accompanied by the husband to the antenatal clinic, while 65 (43.4%) were accompanied to the labor ward by the husbands.

**Table 3:** Birth preparedness among the referrals (N = 140)

†Characteristic	Number (Percentage)
*Making a birth plan*	
Attended antenatal care at least 4 times	132 (94.0)
Had health education on pregnancy and childbirth complications	36 (25.9)
Made a plan of where to deliver from	93 (65.7)
Saved money in case of pregnancy complications	62 (44.3)
Arranged to have a birth companion or attendant during delivery	30 (21.4)

*Ro of husband during antenatal care*	
Accompanied her	60 (42.9)
Took care of domestic chores when she was pregnant	35 (25.0)
Looked after the children at home	58 (41.4)

*Role played by husband during labor*	
Provided transport or gave money for transport	65 (43.4)
Accompanied patient to labor unit	96 (68.6)
Stayed home with children	26 (18.6)
Bought baby/s clothes	62 (44.3)
Got some other person to take care of the home during the mother's absence	58 (41.4)

*Paying for the costs of transport and hospital care*	
Used saved own money	62 (44.3)
Husband provided the money	36 (25.7)
Other relative provided money	42 (30.0)

*Key: (†) Respondents gave multiple responses*.

Table 4 shows the factors associated with having a birth plan. On univariable analysis, age group of adolescents versus older women (Odds ratio (OR) 1.04 (95% confidence limits (CI) 1.0-1.17), Primigravidae compared to multigravidae OR 1.3, (95% CI 1.1-1.6), education level secondary or higher versus primary level or none (OR 2.2 95%CI (1.3-3.6), formal occupation versus informal occupations OR 1.5 95%CI (1.1-2.0) and age of spouse above 25 years compared to 24 years or younger (OR 1.1 95% CI(1.0-1.2), education level of spouse of secondary or higher versus primary level or none, OR 2.0 95% CI (1.3-3.2), and formal occupation versus informal occupation of spouse, OR 1.5 95%CI (1.1-2.0) were associated with having a birth plan. On multivariable analysis, Primigravidae compared to multigravidae, OR 1.8 95%CI (1.0-3.0), education level of spouse of secondary or higher versus primary level or none, OR 3.8 95%CI (1.2-11.0), formal occupation versus informal occupation of spouse, OR 1.6 95%CI (1.1-2.5), presence of pregnancy complications OR 1.4 95%CI (1.1-2.0) and the anticipated mode of delivery of caesarean section versus vaginal delivery, OR 1.6 95%CI (1.0-2.4) were associated with having a birth plan.

**Table 4:** Factors associated with birth preparedness and complication readiness (N = 140)

Characteristic	Crude Odds ratios with confidence interval	Adjusted odds ratios with confidence interval
Age (Adolescents versus older women)	1.04 (1.0-1.17)	0.9 (0.7-1.0)

Parity (Primigravida versus multigravida)	1.0 (0.9-1.3)	1.8 (1.0-3.0)

Age of spouse (Above 25 years versus 24 years or young)	1.0 (1.10-1.2)	1.1 (0.9-1.2)

Education level (Secondary level or higher level versus None or primary level)	2.2 (1.3-3.6)	3.8 (1.2-11.0)

Occupation (Formal versus informal or no employment)	1.5 (1.1-2.0)	2.3 (1.1-4.8)

Marital status	1.5 (0.6-4.0)	-

Occupation of spouse	1.8 (1.2-2.7)	1.6 (1.1-2.5)

Education level of spouse (Secondary level or higher level versus None or primary level)	2.0 (1.3-3.2)	0.7 (0.3-1.9)

Pregnancy complication (Present versus absent)	4.2 (2.0-8.9)	1.4 (1.1-2.0)

Number of times attended antenatal care (four versus more than four)	0.3 (0.0-2.2)	-

Anticipated mode of delivery (caesarean section versus vaginal delivery)	2.2 (1.6-3.0)	1.6 (1.0-2.4)

## Discussion

Antenatal attendance provides an opportunity to inform and educate pregnant women about pregnancy, childbirth and care of the newborn, and therefore enable pregnant women acquire information on danger signs of pregnancy or childbirth. It is anticipated that from antenatal care, women are assisted to develop a birth plan that ensures birth preparedness and readiness in the eventuality of pregnancy or childbirth complications [3,4]. Such a birth plan is expected to assist women in making choices that would contribute to good pregnancy outcome. Our study shows that parity, age of spouse, education level, occupation of spouse, presence of pregnancy complications and the anticipated mode of delivery were associated with having a birth plan. Educated women have better pregnancy outcome compared with uneducated women, possibly since they are better informed, are likely to make better choices, are more likely to develop and implement a birth plan, and are more socially or financially empowered to make the necessary decisions in case of obstetric emergencies [2]. Information, education and counseling plays a vital role in prevention of maternal death. This it does by making the pregnant women (and their partners) aware of the sequence of events from late recognition of danger signs, through delays in seeking care to delays in receiving prompt care. An appropriate programme of health literacy or behavior change communication, such as implementing a birth plan, can circumvent this sequence.

Optimal management of pregnancy, labor and childbirth ensures maternal survival by ensuring that pregnant women (as well as women in labor and their newborns) have access to life saving interventions for managing obstetric and newborn complications [9,10]. In case this care is unavailable at a peripheral facility, mothers are referred to a tertiary facility where care may be obtained. This process inevitably leads to delays in receiving prompt appropriate care. Such delays may result from failure to recognize the need to refer the mother in time, unavailability of transport, failure to meet transport costs, or absence of someone to accompany the referred patient. Once implemented, the birth plan is critical in addressing the first and second of the three delays to receive prompt care in pregnancy and childbirth complications.

Our findings are in agreement with others [11,12] that many patients are admitted when they already have life threatening complications. This is a reflection of the quality of antenatal care at peripheral units (where such complications may be identified early), the quality of obstetric care at the referring units and the efficiency of the referral system. The delays to access care for referrals may be due to problems of geographical access to the hospital, as the district is hilly and many homesteads are inaccessible with motorized transport. The finding that many of the referrals were in critical condition at admission suggests possible delays in making the decision to refer (possibly due to difficulty in diagnosis), delays in reaching the referral hospital or poor quality of care at the referring health facility. Indeed, diagnostic delays and misdiagnosis are responsible for many of the near-miss mortality and are common among emergency obstetric referrals [13,14]. Awareness of the danger signs of obstetric complications is the essential first step in accepting appropriate and timely referral to obstetric and newborn care. Studies that have assessed the availability of a birth plan in pregnant women indicate that opportunity for developing and implementing one are often missed. In a study from Nigeria, 61% of the pregnant women studied made adequate preparations for delivery while only 4.8% were ready for emergency/complication [15], recommending that greater emphasis should be placed on education about emergency/complication readiness during antenatal care. In a study from Kenya [16], 87.3% of the respondents were aware of their expected date of delivery, 84.3% had set aside funds for transport to hospital during labour while 62.9% had funds for emergencies, 67% knew at least one danger sign in pregnancy, while only 6.9% knew of three or more danger signs.

During antenatal care, only 60 (42.9%) mothers reported having been accompanied by spouses, while for 58 (41.4%) the spouse remained at home while looking after the home and children. Thirty five mothers (25.0%) reported that their spouses helped them with household chores during the antenatal period. During labor, 96 mothers (68.6%) were accompanied by their spouses. Apparently, the women who had a birth plan were more likely to be accompanied by the spouses to health facilities during antenatal care and to the labor ward during labor. They were also more likely to report more support from spouses in looking after children or assistance with household chores during pregnancy. Our findings are similar to those from a study in Northern Uganda [17] which found that several men were actively involved in birth prepareness and complication readiness when their spouses were pregnant or in labor. In the Northern Uganda study [17], men who were knowledgeable of ANC services, obtained health information from a health worker and whose spouses utilized skilled delivery at last pregnancy were more likely to accompany their spouses at ANC. This finding suggests that providing information to male partners of pregnant women attending antenatal care might increase their involvement and participation. Prenatal male involvement has been associated with positive outcomes for the mother and baby, which include more antenatal care visits, cessation of smoking and alcohol consumption, participation in high-risk behavior reduction strategies to prevent vertical HIV transmission and more birth preparedness in case of pregnancy complications [7,8,18-22]. Unfortunately, in most studies, male partner involvement in maternal and child health is still low in many sub-Saharan African countries.

In our assessment, we acknowledge several limitations: this study was hospital-based among referred patients, such that results are not generalizable to the community. Secondly, the questions about the birth plan were inquired into after delivery, which may create some bias. Ideally, they should be asked before delivery. Thirdly, presence of obstetric complications may influence the acquisition and therefore availability of a birth plan. Indeed, some women may recall or provide information about the birth plan selectively, depending on the delivery experience or pregnancy outcome. Fourthly, information about spouses' role in birth readiness and complication readiness were asked after delivery and after complications had occurred. However, this was the objective of the study, that is, assessing the role played by spouses in birth plan and complication readiness. There is no way one can verify that the responses were not the socially desirable responses, more so considering that the interviews were conducted at the a health facility. The interviews were conducted in absence of spouses to avoid biased responses. Despite the limitations, we believe the study provides relevant information on birth preparedness and complication readiness for women in rural areas, and identify missed opportunities for interventions to improve emergency obstetric care.

Antenatal care represents a window of opportunity for information, education and communication to pregnant women so that they are well make appropriate choices especially when they are in danger. However this opportunity is often missed [23,24]. This problem is compounded by the inadequate health care system characterized by misplaced priorities, inaccessibility of essential health information to the women most affected, physical as well as economic and geographical distance separating health services from most women, delay to receive adequate and appropriate care [25]. Others are lack of minimal life-saving equipment at the first referral level; the lack of equipment, personnel, and know-how even in referral hospitals. Our findings are in agreement with those in Nigeria [26] that factors influencing maternal health services utilization operate at various levels - individual, household, community and state, and depending on the indicator of maternal health services, the relevant determinants vary.

Observational studies suggest that including men in reproductive health interventions can enhance positive health outcomes. Where pregnant women and their male partners are given health education together, there is a greater net impact on maternal health behaviors (such as healthcare seeking) compared to educating the women alone [27]. Education and health services provided during the antenatal period have the potential to reduce pregnancy and delivery complications and improve birth outcomes in resource-poor settings [28,29]. Women's ability to seek health care or implement lessons learned from health education interventions (by developing their own birth plans) is often determined by the household head, who usually is the husband [30,31]. While the critical role that male partners play in women's reproductive health has been recognized for several years, more attention needs to be focused on involving men in reproductive health education interventions. Men can influence health care utilization during pregnancy and thereby the outcome of an obstetric emergency [32-34] by contributing to development of the birth plan. Indeed, in a study in Northern Uganda [17], men who were knowledgeable of antenatal services and whose spouses utilized skilled delivery at last pregnancy were more likely to accompany their spouses at antenatal care and possibly for delivery.

## Conclusion

The opportunity for developing and implementing a birth plan is often missed such that pregnant women are ill-equipped to make appropriate choices in case of pregnancy complications. Parity, maternal age, education level, age and occupation of spouse and presence of pregnancy complications were associated with having a birth plan.

## Competing interests

The authors declare that they have no competing interests.

## Authors' contributions

DKK and OK conceptualized the study. DKK, OK and MOO designed the study instrument. MOO piloted the study instruments and collected the data. DKK and OK conducted the data analysis. DKK wrote the first draft of the manuscript. All co-authors contributed to revision of the subsequent drafts and approved the final version of the manuscript.

## References

[B1] Hogan MC Foreman KJ Naghavi M Ahn SY Wang M Makela SM Lopez AD Lozano R Murray CJ Maternal mortality for 181 countries, 1980-2008: a systematic analysis of progress towards Millennium Development Goal 5 Lancet 2010 375 9726 1609 23 10.1016/S0140-6736(10)60518-1 20382417

[B2] Uganda Bureau of Statistics (UBOS) and ORC Macro Uganda Demographic and Health Survey 2006 2006 Entebbe, Uganda and Calverton, Maryland, USA: Uganda Bureau of Statistics and ORC Macro

[B3] McPherson RA Khadka N Moore JM Sharma M Are birth-preparedness programmes effective? Results from a field trial in Siraha district, Nepal Journal of Health Population and Nutrition 2006 24 4 479 88 PMC3001152 17591345

[B4] JHPIEGO. Maternal and Neonatal health (MNH) Program Birth preparedness and complication readiness. A matrix of shared responsibilities Maternal and Neonatal Health 2001 23 31

[B5] Kaye D Mirembe F Aziga F Namulema B Maternal mortality and associated near-misses among emergency intrapartum obstetric referrals in Mulago Hospital, Kampala, Uganda East African Medical Journal 2003 80 3 144 9 12762430 10.4314/eamj.v80i3.8684

[B6] Babalola S Fatusi A Determinants of use of maternal health services in Nigeria--looking beyond individual and household factors BMC Pregnancy Childbirth 2009 9 43 10.1186/1471-2393-9-43 19754941 PMC2754433

[B7] Iliyasu Z Abubakar IS Galadanci HS Aliyu MH Birth preparedness, complication readiness and fathers' participation in maternity care in a northern Nigerian community African Journal of Reproductive Health 2010 14 1 22 20695136

[B8] Odimegwu C Adewuyi A Odebiyi T Aina B Adesina Y Olatubara O Eniola F Men's role in emergency obstetric care in Osun state of Nigeria African Journal of Reproductive Health 2005 9 3 59 71 10.2307/3583412 16623190

[B9] Ronsmans C Filippi V Beyond the numbers. Reviewing maternal deaths and complications to make pregnancy safer: reviewing severe maternal morbidity and learning from women who survive life threatening complications 2004 Geneva: World Health Organisation

[B10] Koblinsky MA Beyond maternal mortality-magnitude, interrelationship, and consequences of women's health, pregnancy related complications and nutritional status on pregnancy outcomes International Journal of Gynecology and Obstetrics 1995 48 S21 32 7672173 10.1016/0020-7292(95)02322-4

[B11] Adisasmita A Deviany PE Nandiaty F Stanton Ronsmans C Obstetric near miss and deaths in public and private hospitals in Indonesia BMC Pregnancy Childbirth 2008 8 10 10.1186/1471-2393-8-10 18366625 PMC2311270

[B12] Oladapo OT Sule-Odu AO Olatunji OA Daniel OJ "Near-miss" obstetric events and maternal deaths in Sagamu, Nigeria: a retrospective study Reproductive Health 2005 2 9 10.1186/1742-4755-2-9 16262901 PMC1291401

[B13] Filippi V Ganaba R Baggaley RF Marshall T Storeng KT Sombié I Health of women after severe obstetric complications in Burkina Faso: a longitudinal study Lancet 2007 370 9595 1329 1337 10.1016/S0140-6736(07)61574-8 17933647

[B14] Onwudiegwu U Ezechi OC Emergency obstetric admissions: late referrals, misdiagnoses and consequences Journal of Obstetrics and Gynecology 2001 21 6 570 5 10.1080/01443610120085492 12521770

[B15] Onayade AA Akanbi OO Okunola HA Oyeniyi CF Togun OO Sule SS Birth preparedness and emergency readiness plans of antenatal clinic attendees in Ile-ife, Nigeria Nigerian Postgraduate Medical Journal 2010 17 1 30 9 20348980

[B16] Mutiso SM Qureshi Z Kinuthia J Birth preparedness among antenatal clients East African Medical Journal 2008 85 6 275 83 18817024 10.4314/eamj.v85i6.9625

[B17] Tweheyo R Konde-Lule J Tumwesigye NM Sekandi JN Male partner attendance of skilled antenatal care in peri-urban Gulu district, Northern Uganda BMC Pregnancy and Childbirth 2010 10 53 10.1186/1471-2393-10-53 20846369 PMC2946269

[B18] Roth DM Mbizvo MT Promoting safe motherhood in the community: the case for strategies that include men African Journal of Reproductive Health 2001 5 10 21 10.2307/3583426 12471909

[B19] Msuya SE Mbizvo EM Hussain A Uriyo J Sam NE Stray-Pedersen B Low male partner participation in antenatal HIV counseling and testing in northern Tanzania: implications for preventive programs AIDS Care 2008 20 700 709 10.1080/09540120701687059 18576172

[B20] Katz DA Kiarie JN John-Stewart GC Richardson BA John FN Farquhar C Male perspectives on incorporating men into antenatal HIV counseling and testing PLoS One 2009 4 e7602 10.1371/journal.pone.0007602 19881884 PMC2765726

[B21] Martin LT McNamara MJ Milot AS Halle T Hair EC The effects of father involvement during pregnancy on receipt of prenatal care and maternal smoking Maternal and Child Health Journal 2007 11 595 602 10.1007/s10995-007-0209-0 17557201

[B22] Orne-Gliemann J Desgrees-Du-Lou A The involvement of men within prenatal HIV counselling and testing. Facts, constraints and hopes AIDS 2008 22 2555 7 10.1097/QAD.0b013e32831c54d5 19005286

[B23] Anya SE Hydara A Jaiteh LE Antenatal care in The Gambia: missed opportunity for information, education and communication BMC Pregnancy Childbirth 2008 8 9 10.1186/1471-2393-8-9 18325122 PMC2322944

[B24] Magoma M Requejo J Campbell OM Cousens S Filippi V High ANC coverage and low skilled attendance in a rural Tanzanian district: a case for implementing a birth plan intervention BMC Pregnancy Childbirth 2010 10 13 10.1186/1471-2393-10-13 20302625 PMC2850322

[B25] Sundari TK The untold story: how the health care systems in developing countries contribute to maternal mortality International Journal Health Service 1992 22 3 513 528 10.2190/91YH-A52T-AFBB-1LEA 1644513

[B26] Babalola S Fatusi A Determinants of use of maternal health services in Nigeria--looking beyond individual and household factors BMC Pregnancy Childbirth 2009 9 43 10.1186/1471-2393-9-43 19754941 PMC2754433

[B27] Mullany BC Becker S Hindin MJ The impact of including husbands in antenatal health education services on maternal health practices in urban Nepal: results from a randomized controlled trial urban Nepal: results from a randomized controlled trial Health Education Research 2007 22 2 166 176 16855015 10.1093/her/cyl060

[B28] Carroli G Rooney C Villar J How effective is antenatal care in preventing maternal mortality and serious morbidity? An overview of the evidence Pediatric and Perinatal Epidemiology 2001 15 Suppl. 1 1 42 11243499 10.1046/j.1365-3016.2001.0150s1001.x

[B29] Lumbiganon P Appropriate technology: antenatal care International Journal of Gynecology and Obstetrics 1998 63 Suppl. 1 S91 95 10075217 10.1016/s0020-7292(98)00189-1

[B30] Bloom SS Wypij D Das GM Dimensions of women's autonomy and the influence on maternal health care utilization in a north Indian city Demography 2001 38 67 78 10.1353/dem.2001.0001 11227846

[B31] Beegle K Frankenberg E Thomas D Bargaining power within couples and use of prenatal and delivery care in Indonesia Studies in Family Planning 2001 32 130 146 10.1111/j.1728-4465.2001.00130.x 11449862

[B32] Dudgeon MR Inhorn MC Men's influences on women's reproductive health: medical anthropological perspectives Social Science and Medicine 2004 59 1379 1395 10.1016/j.socscimed.2003.11.035 15246168

[B33] Carter M Husbands and maternal health matters in rural Guatemala: wives' reports on their spouses' involvement in pregnancy and birth Social Science and Medicine 2002 55 437 450 10.1016/S0277-9536(01)00175-7 12144151

[B34] Sternberg P Hubley J Evaluating men's involvement as a strategy in sexual and reproductive health promotion Health Promotion International 2004 19 389 396 10.1093/heapro/dah312 15306623

